# Reply to the Letter “Create Guidelines for Characterization of Venom Peptides” from Dr. Volker Herzig

**DOI:** 10.3390/toxins8090253

**Published:** 2016-08-30

**Authors:** Marcus Vinicius Gomez

**Affiliations:** Institute of Education and Research Santa Casa Belo Horizonte-Laboratory of Toxins, Rua Domingos Vieira 590, Belo Horizonte, Minas Gerais 30150-240, Brazil; marcusvgomez@gmail.com

Regarding our paper “PhTx3-4, a Spider Toxin Calcium Channel Blocker, Reduces NMDA-Induced Injury of the Retina”, published in *Toxins* 2016, *8*, doi:10.3390/toxins8030070 [1]. The structure of PhTx3-4 was not correctly assigned. The correct structure of the toxin is SCINVGDFCDGKKDDCQCCRDNAFCSCSVIFGYKTNCRCEVGTTATSYGICMAKHKCGRQTTCTKPCLSKRCKKNHG, which is the same published by Richardson et al. [2] as PnTx3-4. The MW predicted by the amino acids sequence of the toxin is 8419.7, and by Mass Spectrometry (MS) analysis is 8449.6. We do not yet have a precise explanation regarding the differences seen in the MW between the calculated value and the value obtained by MS analysis. The correct structure, described above, makes it possible for other researchers who do not have access to the crude venom of *P. nigriventer* to reproduce the results obtained by us using chemically synthesized or recombinant-produced toxin as far as the arrangement of disulfide bonds corresponds to that of the native toxin. The accession number of PnTx3-4 sequence deposited in SWISS-PROT/TREMBI is P81790. 

We would like to apologize for the lack of due care while checking the proper sequence of the toxin in our paper. The mistake was generated due to a communication flaw between our research group. We totally agree that the existence of small differences on peptide sequences certainly points to distinct toxins. 

Although is fully true that an alteration on a single peptide can dramatically change the pharmacological activity of a toxin (as stated by Dr. Herzig on his letter), it is equally true that peptides with lower levels of similarity can have similar pharmacological activity. For example, ω-aga IIIA and ω-aga IIIB toxins from the spider *Agelenopsis aperta* have 70.1% and 69.4% similarity to PNTx3-4, respectively, and can exert blockage of high-voltage-activated calcium channels in a similar manner to PNTx3-4 [3]. 

We agree with Dr. Volker Herzig, curator of Arachnoserver data base, that if two homologous toxins have slight differences in their toxin sequence, it is not possible to conclude that these toxins will have a similar activity; however, some isoforms of toxins have the same activity at a certain target. Despite the structure differences between ω-PtxIIA and PhTx3-4, they have the same activity as calcium channel blockers on N and P/Q types [4,5,6,7].
